# Editorial: New Insights into Mechanotransduction by Immune Cells in Physiological and Pathological Conditions

**DOI:** 10.3389/fimmu.2022.930362

**Published:** 2022-05-20

**Authors:** Shizuya Saika, Nicholas Veldhuis, David Križaj, Shaik O. Rahaman

**Affiliations:** ^1^ Department of Ophthalmology, Wakayama Medical University, Wakayama, Japan; ^2^ Drug Discovery Biology Theme, Monash Institute of Pharmaceutical Sciences, Monash University, Parkville, VIC, Australia; ^3^ Department of Ophthalmology, University of Utah School of Medicine, Salt Lake City, UT, United States; ^4^ Department of Nutrition and Food Science, University of Maryland, College Park, MD, United States

**Keywords:** mechanotransduction, inflammation, immunity, TRP channels, Piezo channels

Mechanotransduction is the process in which mechanical and physical forces sensed by membrane receptors and/or channels (‘mechanosensors’) are converted into intracellular biochemical signals. This process plays fundamental functions in the regulation of development, immunity, inflammation, neurodegeneration, wound healing, fibrogenesis, pain transmission, and oncogenesis (1–6). Changes in matrix tension, stiffness (or rigidity), compression, and shear as well as cellular contact with neighboring cells and foreign bodies produce intracellular signals by acting on mechanosensors to affect a wide range of physiological or pathological outcomes (7–14, Gunasinghe et al.). Emerging data support a role for substrate tension, compression, and stiffness of the extracellular and intracellular matrix, in numerous cellular processes including gene expression, cell migration, cell proliferation, and differentiation (1–14, Gunasinghe et al.). Despite substantial progress in mechanotransduction field, the molecular pathways whereby mechanical and biochemical signals are integrated to elicit a specific cellular outcome are still poorly understood. The aim of this special Research Topic, which incorporates 6 original articles and 4 reviews - is to highlight the role of mechanotransduction by immune cells under physiological and pathological conditions.

All immune cells including T-cells, B-cells, Natural killer (NK) cells, macrophages/monocytes, neutrophils, and glial cells of peripheral and central nervous systems are subjected to biochemical and mechanical cues when in systemic circulation and in tissues. NK cells contribute to host immune protection against viruses and tumors by mediating target cell killing and secreting various cytokines. Santoni et al., reports findings on the involvement of mechanosensation and mechanotransduction that is mainly mediated by actin cytoskeleton, in the regulation of NK cell adhesion, migration, tissue infiltration and functions. Precise understanding of mechanotransduction in regulation of NK cell function may facilitate the development of nanomaterials tailored for NK cells, which would be important to develop new immunotherapeutic approaches.

Macrophages and neutrophils are mechanosensitive cells that perform diverse functions by sensing and responding to alteration of both physical and biochemical (soluble) cues in their tissue microenvironment. In contrast to the intense focus on soluble cues including cytokines, chemokines, as regulators of immune cell function, the physical microenvironment has traditionally received considerably less attention. Utilizing static and cyclic uniaxial stretch, Atcha et al. reports that the physical forces synergize with soluble signals to modulate macrophage morphology and function, and suggests a role for CD11b and Piezo1 crosstalk in mechanotransduction in macrophages. In this regard, Orsini et al. summarizes a systematic review to discuss the role of mechanosensitive ion channels, including Piezo1 and transient receptor potential vanilloid 4 (TRPV4), and cell adhesion molecules, including integrins, selectins, and cadherins in immune cells under various physiological and pathological conditions. They have highlighted that mechanical cues *via* activation of mechanosensitive ion channels and receptors activate intracellular signaling pathways, including MAPKs, YAP/TAZ, EDN1, NF-kB, and HIF-1α, to make a change in cellular responses. The precise understanding of the mechanisms by which immune cells transduce mechanosensitive cues presents novel targets to treat human diseases. The review by Nguyen et al. discusses recent understanding of cellular processes whereby macrophage TRPV4 plays a role in pathological conditions and inflammation, and the importance of applying unbiased methods including high-throughput and omics methods in future, for a broader understanding of the functional outcomes of TRPV4 activation.

Toft-Bertelsen and MacAulay, summarizes the current knowledge on cell volume regulation to discuss various mechanisms underlying the molecular coupling from cell swelling to TRPV4 channel activation and present the evidence of direct *versus* indirect swelling-activation of TRPV4. We believe the current knowledge discussed in this review will stimulate further research efforts in this area to precisely reveal TRPV4’s role in numerous physiological and pathological conditions.

Matsuyama et al., summarizes the current knowledge on gateway reflex, a process that explains how autoreactive CD4^+^ T cells cause inflammation in tissues harboring blood-barriers, such as the brain and retina, with a special interest on TRPV1 and mechanotransduction.

The role of TRP channels on inflammation during bacterial infections has been well recognized. Kono et al., reports a previously unsuspected role for TRPV1 and TRPV4 in *Streptococcus pneumoniae* nasal colonization and consequent development of pneumococcal disease in a mouse model. This results show that modulating host immune responses by TRPV1/TRPV4 could be a unique strategy against pathogenic bacteria generating local and/or systemic inflammation.

Trabecular meshwork (TM) cells are mechanosensitive cells with phagocytic and immune properties that actively regulate intraocular pressure (IOP). An increase in IOP stimulates actin polymerization *via* stretch-activated TRPV4 channels, which increases tissue resistance to outflow of aqueous humor to further elevate IOP. Yarishkin et al. report that sustained TRPV4 activation induces pacemaking calcium activity by stimulating TRPM4 (Transient Receptor Potential Melastatin 4), a calcium-activated sodium channel. By imbuing mechanotransduction with intrinsic time-dependence downstream from the tensile and compressive impact of IOP, TRPV4-TRPM4 interactions might provide immune regulation and outflow resistance in the anterior eye with an additional layer of signaling complexity.

Pathni et al., reports that activation of Cytotoxic T lymphocytes (CTLs) in the presence of interleukin (IL)-12 leads to differential modulation of the actomyosin and microtubule dynamics at the immune synapse leading to increased mechanical force exertion by CTLs to their targets. This result indicates a potential mechanotransduction *via* which IL-12 can augment the CTL response.

Initial studies probing mechanosensing role of T cells focused on planar hydrogel and elastomer surfaces. However, these approaches have several drawbacks including difficulties in separating mechanical stiffness from alterations in substrate chemistry required to regulate stiffness. Sachar and Kam, reports here the use of magnetically-actuated microscale elastomer pillars to change the stiffness of elastomer pillars, independent of substrate chemistry.

To precisely understand immune signaling and inflammation it is critical to define the mechanisms by which specific mechanotransduction processes occur, and how functional mechanosensing responses are influenced by the local, systemic and time-dependent biochemical factors. We would like to thank all the authors and reviewers who contributed to this special edition, for their time and expertise.

## Author Contributions

All authors listed have made a substantial, direct, and intellectual contribution to the work and approved it for publication.

## Funding

This work was supported by an R01EB024556 grant to SR, the ARC Centre of Excellence in Convergent Bio-Nano Science and Technology to NV, R01EY027920, R01EY031817, P30EY014800 to DK, and unrestricted support from Research to Prevent Blindness to the Moran Eye Institute at the University of Utah.

## Conflict of Interest

The authors declare that the research was conducted in the absence of any commercial or financial relationships that could be construed as a potential conflict of interest.

## Publisher’s Note

All claims expressed in this article are solely those of the authors and do not necessarily represent those of their affiliated organizations, or those of the publisher, the editors and the reviewers. Any product that may be evaluated in this article, or claim that may be made by its manufacturer, is not guaranteed or endorsed by the publisher.
